# Supplementary opinions on alternative cooling technologies in hot climate

**DOI:** 10.1007/s00484-018-1588-1

**Published:** 2018-08-02

**Authors:** Bin Yang, Faming Wang

**Affiliations:** 1 School of Environmental and Municipal Engineering, Xi’an University of Architecture and Technology, Xi’an, China; 2 Department of Applied Physics and Electronics, Umeå University, Umeå, Sweden; 3 Institute of Textiles and Clothing, The Hong Kong Polytechnic University, Hung Hom, Kowloon, Hong Kong China

## Abstract

Some supplementary opinions on alternative cooling technologies for hot climate were presented to contribute towards the comprehensiveness of the review paper reported by Lundgren-Kownacki et al. (Int J Biometeorol 62:401–12, 2018).

The challenges of using air conditioning (AC) in hot climate were comprehensively analyzed in terms of environmental, organizational, socialeconomic, biophysical, and behavioral factors (Lundgren-Kownacki et al. 2018). Possible alternative solutions to AC were summarized including climate-sensitive urban planning and building design (passive strategies), alternative cooling technologies (active strategies), and climate-sensitive attitudes and behavior (adaptation). The authors would like to give some supplementary opinions on alternative cooling technologies used in hot climate, which may contribute towards the comprehensiveness of the review paper by Lundgren-Kownacki et al. (2018).

Two concepts should be clarified, based on the title of the review paper. AC has different types for residential buildings and public buildings. Hot climate mainly includes hot-humid climate and hot-dry climate. Only split air conditioner was reviewed, mainly for Hanoi housing project and some cases in India and Thailand. The use of fan, instead of AC, met the demands of low-income households. Only mentioning this opinion may cause bias. The authors agreed with the findings observed in some developing tropic countries such as India, Brazil, and Southeast Asia countries. However, elevated air speed by an energy-efficient fan (ceiling fan, stand fan, table fan) can work together with less intensified AC (elevated AC setpoint temperature) to achieve energy-efficient thermal comfort. Aforementioned AC includes split air conditioner without fresh air for residential buildings and centralized air conditioning system with fresh air for public buildings. Elevated air speed, which may cause draft under neutral-cool environments (Fanger et al. 1988), is a positive factor to satisfy human thermal comfort under neutral-warm environments. Tropically acclimatized occupants prefer slightly high air movement (Gong et al. 2006). Corresponding threshold value of air speed for indoor environments was elevated (ANSI/ASHRAE, 2013). Fan-aided less intensified AC can effectively avoid the overcooling problem especially for public office buildings (Schiavon et al. 2017). In a hot-humid climate, dehumidification is important especially when AC setpoint temperature is elevated. Heat pipe technology and desiccant cooling technology were used to guarantee dehumidification effects. Task ambient control (TAC) concept was pointed out in the 1990s, which emphasized intensified conditioning of human occupied task area and less intensified conditioning of unoccupied ambient area (Bauman and Arens 1996). In the tropics, the concept can be achieved by personalized ventilation (Fanger 2001) by considering both local cooling and air quality, and personal comfort system (PCS) by considering local fan cooling. Aforementioned topics were explored for several decades and should be added as alternative cooling technologies.

In the review paper authored by Lundgren-Kownacki et al. (2018), some passive cooling strategies such as enhanced natural ventilation, minimize solar heat gain, green structures, and some active cooling strategies such as evaporative cooling in hot-dry climate are well recognized. Some of them, such as district cooling, are still controversial which should be mentioned with caution. Cooling vest with phase change materials was also mentioned, which is one traditional research field in clothing and material field. More recently, the concept of personal thermal management by carefully choosing clothing materials was pointed out by researchers from Stanford University, which developed task control from human occupied area to the human body. Nano-technologies were applied to clothing, which enhances heat preservation or cooling effects of clothing. Metallic nanowire mesh, whose empty space is smaller than infrared wavelength, was coated onto textiles to reflect back infrared radiation towards the human body to reduce heat loss (Hsu et al. 2014). Perspiration-generated water vapor, whose molecule size is much smaller than the spacing, can permeate the spacing to guarantee wearer’s comfort. Compared to normal polyethylene textiles, the specially designed nanoporous polyethylene textile is visibly opaque, infrared transparent textile with good air permeability and water wicking properties, which may be suitable for passive human body cooling (Hsu et al. 2016). Similar studies of nanoporous metalized polyethylene textiles for personal cooling or heating were also performed (Cai et al. 2017; Peng et al. 2018). Based on aforementioned studies, a dual-mode textile for both heating and cooling was developed by using a bilayer thermal emitter embedded inside the infrared transparent nanoporous polyethylene textile (Hsu et al. 2017).
